# Comparing the effectiveness of group-based exercise to other non-pharmacological interventions for chronic low back pain: A systematic review

**DOI:** 10.1371/journal.pone.0244588

**Published:** 2020-12-30

**Authors:** James Lemieux, Vahid Abdollah, Brandyn Powelske, Greg Kawchuk

**Affiliations:** Department of Physical Therapy, Faculty of Rehabilitation Medicine, University of Alberta, Edmonton, AB, Canada; Baylor College of Medicine, UNITED STATES

## Abstract

**Background:**

Low back pain (LBP) is the leading cause of disability worldwide with a substantial financial burden on individuals and health care systems. To address this, clinical practice guidelines often recommend non-pharmacological, non-invasive management approaches. One management approach that has been recommended and widely implemented for chronic LBP is group-based exercise programs, however, their clinical value compared with other non-pharmacological interventions has not been investigated systematically.

**Objective:**

To compare the effectiveness of group-based exercise with other non-pharmacological interventions in people with chronic LBP.

**Methods:**

Four electronic databases were searched by two independent reviewers. Only randomized controlled trials that compared group-based exercise with other non-pharmacological interventions for chronic LBP were eligible. Study quality was assessed using the Cochrane Handbook for systematic reviews of Interventions by two independent reviewers.

**Results:**

Eleven studies were eligible. We identified strong evidence of no difference between group exercise and other non-pharmacologic interventions for disability level and pain scores 3-month post-intervention in people with chronic LBP. We could not find any strong or moderate evidence for or against the use of group-based exercise in the rehabilitation of people with chronic LBP for other time-points and health measurement outcomes. We found no statistically significant differences in disability and quality of life and pain between the group and individual non-pharmacological interventions that included exercise.

**Conclusion:**

With this equivocal finding, group-based exercise may be a preferred choice given potential advantages in other domains not reviewed here such as motivation and cost. Further research in this area is needed to evaluate this possibility.

## Introduction

Low back pain (LBP) is the leading cause of disability globally with a substantial financial burden on individuals, families, communities and governments worldwide [1]. At an individual level, LBP diminishes quality of life by limiting activities of daily living, deteriorating mental health, decreasing life span [2] and inducing financial hardships [3]. Therefore, LBP is thought to be the most costly disability of the working-age population [4]. The nature of LBP is highly prevalent and recurrent: the lifetime occurrence is estimated to be 85%, and ~50% of people will have at least 10 episodes in their lifetime [1].

In addressing chronic LBP, clinical practice guidelines often recommend non-pharmacological and non-invasive management approaches for chronic LBP [3]. Specifically, these guidelines recommend education and exercise as first-line interventions [5–7]. While many randomised controlled trials have provided scientific evidence supporting the benefits of exercise in chronic LBP [8], how to best deliver exercise interventions is less clear. Individual exercise programs are the most widely implemented approach for addressing chronic LBP [9]. In contrast, group exercise-based classes have been found to be beneficial [10–12], but are not as widely used. Group exercise may be an equally effective alternative to individual exercise with potentially lower healthcare costs [8]. The potential for social support and better social interaction in groups should also be considered a potential advantage [8]. With this in mind, group exercise approaches have been recommended by the National Institute of Health and Care Excellence [12].

Given the above, we could not identify any prior systematic reviews that compared group-based exercise to individual non-pharmacological interventions that may include education and/or exercise in people with chronic LBP. Therefore, we conducted this review to evaluate the comparative effectiveness of group-based exercise to other non-pharmacological interventions that may or may not include education and exercise on pain and disability in patients with chronic LBP.

## Methods

In this systematic literature review, we considered group exercise as the intervention and employed the Cochrane Handbook for Systematic Reviews of Interventions [13]. Our reporting was planned according to the Preferred Reporting Items for Systematic Reviews and Meta-Analyses (PRISMA) statement [14].

### Literature search and study selection

A systematic search was conducted on June 26, 2020, using MEDLINE^®^, EMBASE, CINAHL, and Scopus. Search terms were selected through consultation between two rehabilitation experts and a university librarian. References cited within included articles were reviewed to identify additional studies. Two authors (JL and VA) selected studies up until June 26, 2020 that compared group exercise with other forms of intervention programs for people with LBP. Results from each database were uploaded to Covidence (www.covidence.org) and duplicates were excluded after software review.

Group-based exercise programs were defined as a group of three or more participants taking part in an exercise class supervised by a health care provider. A non-pharmacological intervention was defined as one-on-one care between a health care provider and their patient that did not involve pharmaceuticals. The intervention programs were identified using the search terms “group exercise”,” “GLA:D Back”, “group strengthening”, “group physical activity”, or “group strength training”. Low back pain was identified using the search terms “chronic back pain”, “persistent back pain”, “long-standing back pain”, “long-duration back pain”, “long-standing lumbar pain”, “long-duration lumbar pain”, “chronic low back pain”, “persistent low back pain”, “long-standing low back pain”, or “long-duration low back pain”.

### Eligibility criteria

Only peer-reviewed, randomized, controlled trials comparing group-based exercise including strengthening, physical activity, and strength training with other types of non-pharmacologic interventions for chronic LBP were included. We excluded reports related to conference proceedings, specific low back pain diagnoses, case series of fewer than ten subjects, case studies, systematic reviews, and protocol papers.

### Selection of studies

Two investigators (JL and VA) with more than 10 years of cumulative experience in reviewing literature screened all titles and abstracts independently and retrieved the full text of the potentially eligible studies. Disagreements at the titles and abstracts stage were resolved through consensus.

### Data extraction

A standard form (S2 Appendix) was developed to extract data based on published guidelines [15–17]. Data for each study were extracted and cross-checked by two investigators (JL and VA). Disagreements were resolved by a third investigator (GK). The following information was extracted for each study: 1) characteristics of the participants: sample size, age, gender, height, diagnosis, pain duration, location and intensity; 2) inclusion and exclusion criteria; 3) characteristics of the interventions: the type, length of the program, mode of application, frequency and duration of group and individual exercise based physiotherapy; 4) characteristics of the outcomes: pain and disability outcomes measures, follow-up times.

### Methodological quality

The quality of included studies was assessed as outlined by PRISMA, and the Strengthening the Reporting of Observational Studies in Epidemiology (STROBE) guidelines [18]. The quality appraisal focused on seven categories: subject recruitment, examiners, methodology, outcomes, handling of missing data, statistical analysis, and results (S3 Appendix). Two reviewers (JL, VA) conducted critical appraisal separately on each of the papers and decisions were verified through consensus. Practice appraisals and discussion of five full-text papers occurred for calibration before the full review. Studies with a minimum score of 70% were considered to be of high quality and those with a lower score to be of low quality [19].

### Data synthesis and analysis

**Fig 1:**
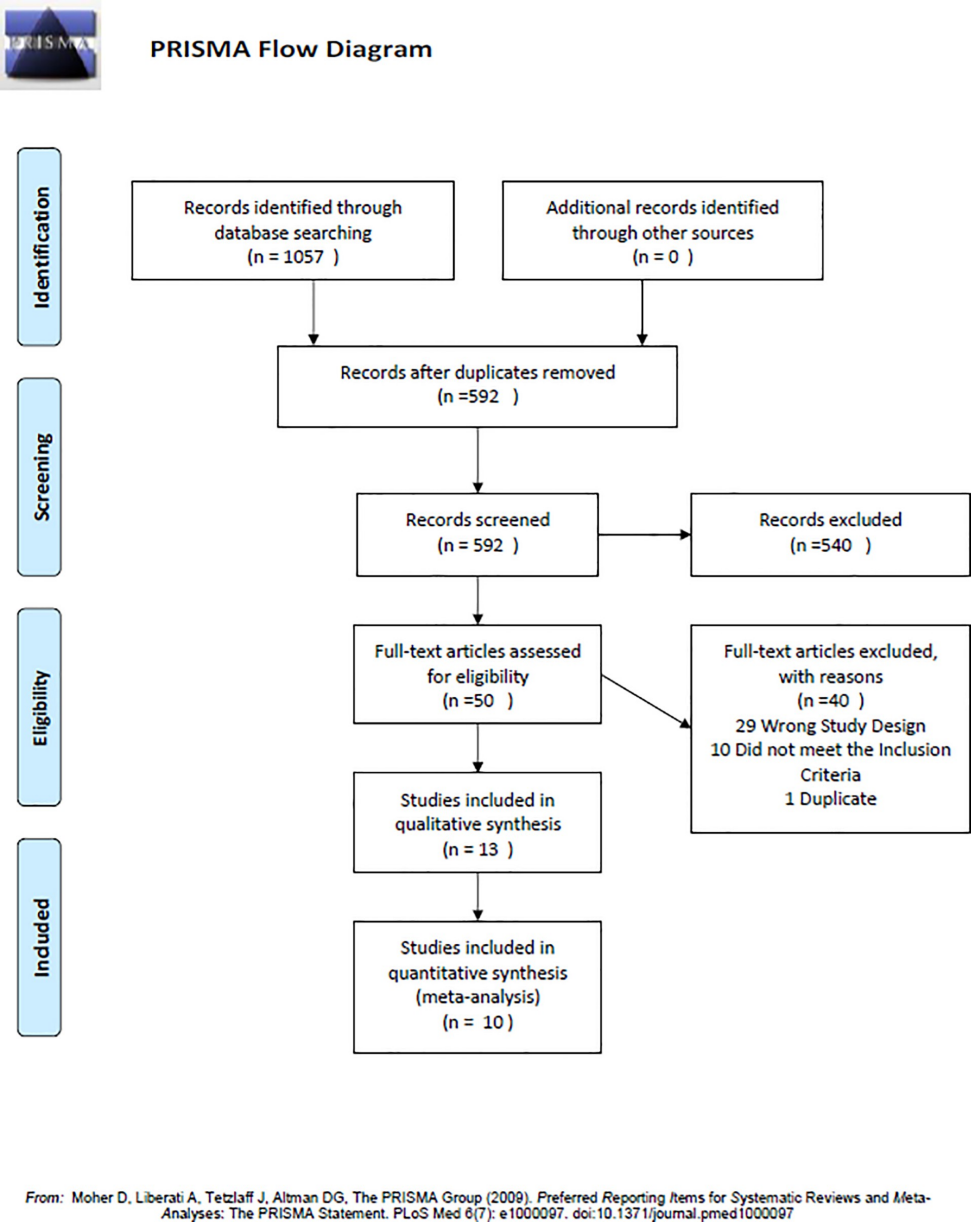
Search strategy guided by the PRISMA flow diagram.

A PRISMA flowchart was constructed to summarise the article selection process (Fig 1) [14]. Agreement between reviewers on article selection at each stage and on the quality appraisal of the included full-text articles was described using percentages. The level of evidence (strong, moderate, limited, no, and conflicting evidence) for the effect of interventions was determined according to the consistency of the research findings and the methodological quality of the included studies [19]. The level of evidence was considered strong if there was more than 75% agreement between at least two high-quality studies and more than two low-quality studies on the outcome of the interest (Table 1) [19].

**Table 1:** Levels of evidence for summary statements and description of criteria adopted a priori to determine the level of evidence [19].

Level	Description
**Strong**	Consistent results (≥75%) from at least 2 high-quality* studies
**Moderate**	1 high-quality* study and consistent findings (≥75%) in 1 or more low-quality studies
**Limited**	Findings in 1 high-quality* study or consistent results (≥75%) among low-quality studies
**No**	No study identified
**Conflicting**	Inconsistent results irrespective of study quality

*Studies with quality scores over 70% were deemed high quality.

The evidence was considered moderate if there was more than 75% agreement between a high-quality study and at least three low-quality studies (Table 1) [19]. The evidence was considered limited if only one high-quality study reported that outcome or at least three out of four low-quality studies (75%) reported the same outcome (Table 1) [19]. The evidence was considered conflicting if there was less than 75% agreement among the studies irrespective of study quality (Table 1) [19].

Summary tables were prepared for participants’ descriptions (Table 2), intervention used (Table 3), quality appraisal scores (Table 4), the level of evidence summary statements and outcomes extracted (Table 5).

**Table 2:** Description of study type and study participants in the included studies.

Authors	Study Type	Recruitment Strategy and Selection Criteria	Number of Subjects and	Participant Age	Diagnosis	Pain
			Groups	(years)		(Duration)
**Daulat** [21]	Permuted Blocks, Single Blinded, Two-arm RCT with 6-month follow-up	Male and female	Spinal Rehabilitation:	Spinal Rehabilitation:	Chronic LBP referred from General Physicians	Median (Interquartile Range):
		Aged 20–75 years,				
						Spinal Rehabilitation:
		Mechanical Chronic LBP >3 months	15♂, 26♀	46.4 ±12.1		
						36.0 (61) Months
		Motivated and willing to attend both the physiotherapy group programmes	Back to Fitness:	Back to Fitness:		
						Back to Fitness:
			16♂, 24♀	43.3 ±12.7		
						21.5(62) Months
**Harris et al.** [27]	Three-arm RCT with	At least 50% sick leave due to unspecific LBP,	Brief Intervention:	Brief Intervention:	Non-specific LBP	Brief Intervention
			43♂,56♀	44.8±9.7		
						12.5±11.3 years
		Aged: 20–60 years, being	Cognitive Behavioral Therapy:	Cognitive Behavioral Therapy:		
		At least 50% employed				Cognitive Behavior Therapy
		Having one of the following International Classification of Primary Care diagnoses for the current sick leave episode				
			31♂,24♀	45.5±9.1		
			Physical Exercise	Physical Exercise:		9.6±10.9 years
						Physical Exercise
			32♂,28♀	44.2±10.6		
						11.5±10.6
**Hurley et al.** [24]	An assessor-blinded, Three-arm RCT trial with and 12-month follow-up	Male and female	Exercise:	Exercise:	Non-specific chronic or recurrent LBP	Exercise:
		Chronic LBP (≥3 Months) or recurrent (≥3 episodes in previous 12 Months)				
		Mechanical LBP with/without radiation to the lower limb				
		Aged 18–65 years				
		No spinal surgery within the past 12 Months				
			24♂, 59♀	45.8±11.1		7±8.0 years
		Deemed suitable by their general practitioner/hospital				
			Walking:	Walking:		Walking:
		consultant to carry out an exercise program				
			24♂, 58♀	46.2±11.3		8.7±9.0 years
		willing to attend an 8-week treatment program of exercise classes				
			Usual Physiotherapy:	Usual Physiotherapy:		Usual Physiotherapy:
			31♂, 50♀	44.2±11.7		7.5±7.9 years
		Access to a telephone (for follow-up support)				
		Fluency in English (verbal and written)				
		Low” or “moderate” levels of PA measured by the IPAQ (<600 metabolic equivalents of the task -minutes/				
		week)				
**Johnson et al.** [20]	Two-arm RCT with 15-month follow-up	Aged 18–65 years	Active intervention	Active intervention	LBP	?
		Consulting General Physicians with LBP between January 2002 and July 2003	45♂, 71♀	47.3±10.9		
			Control:	Control		
			49♂, 69♀	48.5±11.4		
**Lewis et al** [23]	Two-arm RCT	Aged between 18–75 years,	Group exercise	Group exercise	Non-radicular mechanical LBP	Group exercise
						11.1±12.6 years
			14♂, 26♀	46.1±12.7		
		fluency in English,	Individual exercise	Individual exercise		Individual exercise
		LBP >3 months				
			26♂, 14♀	45.7±12.7		
						10.1±9.9 years
**Masharawi & Nadaf** [25]	Single-blinded, pilot, Two-arm RCT with 12-week follow up	Female,	Group Exercise	Group Exercise	Non-specific LBP	Minimum of 12 weeks,
		Aged 45–65 years,				
		LBP > 12 weeks,	20♀	52.4±10.6		
		Able to give informed consent,	Control	Control		
		Understood instructions,	20♀	53.6±9.5		
		Willing to cooperate with the treatment.				
**O'Keeffe et al.** [28]	Pragmatic, Two-arm RCT with 12 months post-randomisation	Chronic LBP	Group-based exercise and education intervention	Group-based exercise and education intervention	Chronic LBP	Median: 60 months
			30♂, 70♀	47.0±13.2		
			Cognitive functional therapy	Cognitive functional therapy		
			24♂, 82♀	50.6±14.9		
**Ryan et al.** [26]	Single-blinded, Two-arm RCT with 3-month follow up	Male and female	Education + Exercise:	Education + Exercise:	Non-specific LBP	Education + Exercise:
		Aged 18–65 years	6♂, 14♀	45.2±11.9		28.1±20.4
		Pain >3 Months				
			Education:	Education:		Education:
		No history of surgery				
			7♂, 11♀	45.5±9.5		39.3±26.2
**Sahin et al.** [22]	Two-arm, RCT 3-month follow-up	Non-specific LBP >12 weeks	Back school:	Back school:	Non-specific LBP	Back school:
			18♂, 55♀	47.2±11.2		6.5±7.3 months
		without neurological deficits	Control:	Control:		Control:
			16♂, 57♀	51.4±9.6		7.3±6.5 months
**Sherman et al.** [29]	Three-arm RCT with 26-week follow-up	Aged 20–64 years	Yoga	Yoga	LBP	Most experienced back pain more than 1 year before the study,
				44±12.0		
			11♂, 25♀*	Group exercise		
			Group exercise			
		Had visited a primary care provider for treatment of LBP 3 to 15 months before the study	13♂, 22♀	42±15.0		
			Self-Care Book	Self-Care Book		Two-thirds of participants reported pain lasted for more than 1 year.
			10♂, 20♀			
				45±11.0		
**Carr et al.** [30]	Two-arm RCT with 12-month follow-up	Mechanical LBP lasting at least six weeks	Individual Physiotherapy	Individual Physiotherapy	Mechanical LBP	Individual Physiotherapy
						54%>6 months
			45♂, 74♀	42.5±11.2		
						46%<6 months
			Group Exercise	Group Exercise		
						Group Exercise
						65%>6 months
			49♂, 69♀	42.0±10.6		
						35%<6 months

Abbreviations and symbols: RCT: Randomized Control Trial; LBP: Low Back Pain; ♂: males; ♀: females.

*Gender percentages are converted to a number.

**Table 3:** Description of the intervention used in the included studies.

Authors	Groups	Intervention	Duration	Metric	Data Collection Timepoints
**Daulat** [21]	Experimental	Group multimodal exercise therapy + one-to-one education and/or manual therapy sessions	Six 1-hour treatment sessions over a 3-month period	Functional Rating Index	BL
				NPRS	
				EQ- 5D-5L	
	Control	General exercise sessions using a circuit-based exercise format + weekly group education sessions at the end of the exercise period.			POI
					6M POI
				Participant Satisfaction Reporting Scale	
				Group interviews	
**Harris et al.** [27]	Brief cognitive intervention	Brief cognitive, clinical examination program based on a non-injury model addressing pain and fear avoidance, where return to normal activity and work is the main goal.	two sessions over a period of 5 days with the choice of two booster sessions.	Increased work participation	BL
				ODI	
				Hospitality Anxiety and Depression Scale	
				Subjective Health Complaints Inventory	
	Brief cognitive intervention + Cognitive-behavioural treatments	Cognitive-behavioural treatment manual adopted from the CINS trial [31]	7 session at 90min for a total of 10.5 hours over a 3-month period		Monthly POI up to 12 months
				Utrecht Coping List	
				Instrumental Mastery-Orientated Coping	
	Brief cognitive intervention + physical group exercise	Strength and endurance training + relaxation	90 min, Three times/week over a 3-month period		
				Fear-Avoidance Beliefs Questionnaire	
**Hurley et al.** [24]	Walking	Walking	10-min walk at least 4 days per week proceed to	ODI	BL
				NPRS	
				Fear Avoidance Beliefs Questionnaire-PA subscale	
			30 min of moderate-intensity PA for 5 days per week at week 5 for a total of 8 weeks		
				Back Beliefs Questionnaire	3M POR
					6M POR
				International Physical Activity Questionnaire	
					12M POR
	Exercise class	A programme of progressive or graded exercises + a back-care education message	1-hour weekly class up to 8 weeks	Exercise Self-efficacy Questionnaire	
				Readiness to Change Questionnaire	
	Usual physiotherapy	Individualized education/advice, exercise therapy + manipulative therapy	?		BL
				Patient Satisfaction Questionnaire	
					3M POR
**Johnson et al.** [20]	Active intervention	Booklet and audiocassette + community-based treatment program (problem-solving, pacing and regulation of activity, challenging distorted cognitions about activity and harm, and helping patients to identify helpful and unhelpful thoughts about pain and activity)	Eight 2-hour group sessions over a 6-week period	VAS	BL
				RMDQ	
				General Health Questionnaire	3M POI
					9M POI
				EQ-5D	15M POI
	Control	Booklet and audiocassette	None		
**Lewis et al** [23]	Exercise class	10 station exercise class involving aerobic exercises, spinal stabilization exercises, and manual therapy	8 treatments over 8 weeks	Lumbar flexion	POI
				Lumbar extension	
				Side flexion	6M POI
					12M POI
				Straight leg raising test	
	Individual treatment	One-to-one intervention, 30 minutes of manual therapy (mobilizations to the spine) and spinal stabilization exercises			
				Quebec back pain disability scale	
**Masharawi & Nadaf** [25]	Group exercise	10 repetitions of 10 exercises aimed at improving lumbar mobility/flexibility and stability	45 min group exercise session twice a week, over 4 weeks, Thereafter, monthly meetings took place to review and reinforce program consistency.	VAS	BL
				RMDQ	
				Flexion ROM	4W POI
				Extension ROM	8W POI (only intervention group)
				Left and right rotation ROM	
	Control group	Waitlist			
**O'Keeffe et al.** [28]	Group-based exercise and education	Three components to the intervention: 1) pain education; 2) exercise; and 3) relaxation.	Up to six classes over 6–8 weeks, each lasting ~1 hour and 15 min, with up to 10 participants in each class.	ODI	BL
				Numerical Rating Scale	
				Fear-avoidance using the physical activity subscale of the Fear Avoidance Beliefs Questionnaire	
	Cognitive functional therapy	Comprehensive one-to-one interview and physical examination by physiotherapists.	Length varied in a pragmatic manner based on the clinical progression of participants.	Coping subscale of the Coping Strategies Questionnaire	
				Pain Self-Efficacy Questionnaire	
				Nordic Musculoskeletal Questionnaire	6M POR
				Örebro musculoskeletal screening questionnaire	12M POR
		Three components to the intervention: 1) cognitive component: making sense of pain; 2) exposure with ‘control’; and 3) lifestyle change, which have been described in detail elsewhere			
				Subjective Health Complaints Inventory	
				Depression, Anxiety and Stress Scale	
				Patient Satisfaction Questionnaire	
**Ryan et al.** [26]	Education and exercise group	Pain biology education + “The Back Book” + group exercise (Back to the Fitness exercise program, circuit-based, graded, aerobic exercise with some core stability exercises)	six classes, once a week for six weeks	RMDQ	BL
				NPRS	
				Repeated sit-to-stand test	
				Fifty-foot walk test	
	Education only group	Pain biology education cognitive behavioural intervention +		5-min walk test	POI
				Tampa Scale of Kinesiophobia-13	3M POI
			One session lasted 2.5 hrs	Pain self-efficacy questionnaire	
				Step-count for 1W	
		“The Back Book”			
**Sahin et al.** [22]	Back school + Exercise + Physical therapy	Didactic and practical	1 hour, 2 times a week for 2 weeks	VAS	BL
		training			
		Lumbar flexion exercises	5 times a week for 2 weeks		
		Lumbar extension			
		Lumbar stretching exercises, and strengthening exercises			
		Transcutaneous electrical nerve stimulation, ultrasound, and hot pack			
				ODI	3M POI
	Control	Lumbar flexion exercises			
		Lumbar extension			
		Lumbar stretching exercises, and strengthening exercises			
		Transcutaneous electrical nerve stimulation, ultrasound, and hot pack			
**Sherman et al.** [29]	Yoga	Yoga session + auditory compact discs to guide them through the sequence of postures with the appropriate mental focus	75 min weekly for 12 weeks	Telephone interviews	BL
	Conventional therapeutic exercise classes	short educational			
		talk + exercise class (7 aerobic exercises and 10 strengthening exercises that emphasized leg, hip, abdominal, and back muscles)			
					6W POR
				RMDQ	
				Short Form-36 Health Survey	
					12W POR
					26W POR
	Self-care book.	The Back-Pain Help book	?		
**Carr et al.** [30]	Back to Fitness Program	Low impact aerobics, strengthening and stretching exercises for the main muscle groups, and relaxation + A cognitive-behavioural approach underpinned messages	8 hrs. over a 4-week period	RMDQ	3M
	Physiotherapy	One (or a combination) of McKenzie exercises, strengthening exercises, stretching exercises, spinal stabilizations, other exercises, manipulation, mobilizations, traction, Short wave diathermy, ultrasound, interferential, TENS, other treatment (including massage, heat, laser, advice/education).	?	SF12	
				EQ5D	12M
				Pain Self-Efficacy Scale	

BL: baseline; min: minutes, hrs.: hours, POI: post-intervention; POR: post-randomization, W: Week; M: Month; VAS: Visual Analogue Scale; ODI: Oswestry Disability Index; NPRS: Numerical Pain Rating Scale; RMDQ: Roland and Morris Disability Questionnaire; ROM: range of motion.

**Table 4:** Quality appraisal of the studies included.

Authors	Recruitment	Examiners	Methodology	Outcomes	Missing Data	Statistical Analysis	Results	Overall Score	Overall Score
	/7	/4	/5	/2	/8	/5	/2	/33	(%)
Daulat [21]	5	1	5	2	2	2	1	18	56%
Harris et al. [27]	6	2	2	2	5	3	1	21	66%
Hurley et al. [24]	6	2	4	1	4	3	2	22	69%
Johnson et al. [20]	6	0	4	2	6	4	1	23	72%
Lewis et al [23]	6	2	3	2	2	4	1	20	63%
Masharawi & Nadaf [25]	6	1	4	1	6	4	1	23	72%
O’keeffe [28]	5	4	5	2	4	5	2	27	82%
Ryan et al. [26]	7	0	3	1	4	4	2	21	66%
Sahin et al. [22]	5	2	4	1	5	5	2	24	75%
Sherman et al. [29]	6	3	4	1	4	4	2	24	75%
Carr et al. [30]	6	2	4	2	5	4	1	24	75%

Overall score: the sum of all scores.

**Table 5:** Levels of evidence for summary statements for each intervention.

Level of evidence	From n studies	Changes	Data Collection Time-point	Groups compared
**Pain (Numeric pain Rating Scale and Visual Analogue Scale)**				
Limited	1 [21]	No difference	Post-intervention	Exercise Group vs. Individual Treatment
Limited	1 [25]	A lower score for Group Exercise	4-week post-intervention	Group Exercise vs. Control group
Conflicting	3 [20, 22, 26]	Inconsistent	3-month post-intervention	Exercise &Education vs. Education Group Exercise vs. Pain Biology
				Back school + Exercise + Physical therapy vs. Control
Limited	1 [21]	No difference	6-month post-intervention	Exercise Group vs. Individual Treatment
Limited	1 [26]	A lower score for Group Exercise	0, 3, & 6-month post-intervention	Exercise &Education vs. Education
Limited	1 [20]	No difference	9-month post-intervention	Active Intervention vs. Control
Limited	1 [20]	No difference	15-month post-intervention	Active Intervention vs. Control
Limited	1 [24]	No difference	3-month post-randomization	Walking vs. Exercise Class vs. Usual Physiotherapy
Moderate	2 [24, 28]	No difference	6-month post-randomization	Walking vs. Exercise Class vs. Usual Physiotherapy
				Group-based exercise + education vs. Cognitive functional therapy
Moderate	2 [24, 28]	No difference	12-month post-randomization	Walking vs. Exercise Class vs. Usual Physiotherapy
				Group-based exercise + education vs. Cognitive functional therapy
**Disability**				
Limited	1 [23]	A lower score for individual intervention	Post-intervention	Group Intervention vs. Individual Intervention
				Group Exercise vs. Pain Biology
Limited	1 [25]	A lower score for Group Exercise	4-week post-intervention	Group Exercise vs. Control group
Strong	4 [20, 22, 26, 30]	No difference	3-month post-intervention	Active Intervention vs. Control
				Group Exercise vs. Pain Biology
				Back school + Exercise + Physical therapy vs. Control
				Group Exercise vs. Individual Physical Therapy
Limited	1 [23]	A lower score for individual intervention	6-month post-intervention	Group Intervention vs. Individual Intervention
Limited	1 [26]	No difference	0, 3-month, & 6-month post-intervention	Exercise &Education vs. Education
Limited	1 [20]	No difference	9-month post-intervention	Active Intervention vs. Control
Limited	3 [23, 27, 30]	Inconsistent	12-month post-intervention	Walking vs. Exercise Class vs. Usual Physiotherapy
				Group Exercise vs. Individual Treatment
				Group Exercise vs. Individual Physical Therapy
Limited	1 [20]	No difference	15-month post-intervention	Active Intervention vs. Control
Limited	1 [29]	Lower scores in Yoga group	6-week post-randomization	Yoga vs. Conventional Therapeutic Exercise Classes vs. Self-care Book
Conflicting	2 [24, 29]	Inconsistent	3-month post-randomization	Walking vs. Exercise Class vs. Usual Physiotherapy
				Yoga vs. Conventional Therapeutic Exercise Classes vs. Self-care Book
Conflicting	3 [24, 28, 29]	Inconsistent	6-month post-randomization	Walking vs. Exercise Class vs. Usual Physiotherapy
				Yoga vs. Conventional Therapeutic Exercise Classes vs. Self-care Book
				Walking vs. Exercise Class vs. Usual Physiotherapy
Limited	1 [28]	A lower score for Cognitive functional therapy	12-month post-randomization	Group-based exercise + education vs. Cognitive functional therapy
**Lumbar Spine Flexibility (Flexion, Extension, and Lateral Flexion)**				
Limited	1 [23]	No difference	Post-intervention	Exercise Class vs. Individual Treatment
				Group Intervention vs. Individual Intervention
Limited	1 [25]	A higher score for Group Exercise	4-week post-intervention	Group Exercise vs. Control group
Limited	1 [25]	A higher score for Group Exercise	8-week post-intervention	Group Exercise vs. Control group
Limited	1 [23]	Higher ROM for lumbar extension and side bending and no difference for flexion	6-month post-intervention	Exercise Class vs. Individual Treatment
				Group Intervention vs. Individual Intervention
Limited	1 [23]	No difference	12-month post-intervention	Exercise Class vs. Individual Treatment
**Fear Beliefs**				
Limited	1 [26]	No difference	0, 3-month, & 6-month post-intervention	Exercise &Education vs. Education
Limited	1 [24]	No difference	3-month post-intervention	Walking vs. Exercise Class vs. Usual Physiotherapy
Limited	1 [24]	No difference	6-month post-intervention	Walking vs. Exercise Class vs. Usual Physiotherapy
Limited	2 [24, 27]	No difference	12-month post-intervention	Brief Intervention vs. Brief Intervention + Cognitive Behavioral Therapy vs. BI + Physical Group Exercise
				Walking vs. Exercise Class vs. Usual Physiotherapy
Limited	1 [26]	No difference	0, 3-month & 6-month post-randomization	Walking vs. Exercise Class vs. Usual Physiotherapy
Limited	1 [28]	No difference	6-month post-randomization	Group-based exercise + education vs. Cognitive functional therapy
Limited	1 [28]	No difference	12-month post-randomization	Group-based exercise + education vs. Cognitive functional therapy
**Health Surveys**				
Limited	1 [21]	No difference	Post-intervention	Exercise Group vs. Individual Treatment
Strong	2 [20, 30]	No difference	3-month post-intervention	Active Intervention vs. Control
				Group Exercise vs. Individual Physical Therapy
Limited	1 [21]	No difference	6-month post-intervention	Exercise Group vs. Individual Treatment
Limited	1 [20]	No difference	9-month post-intervention	Active Intervention vs. Control
Limited	1 [30]	No difference	9-month post-intervention	Active Intervention vs. Control
Limited	1 [20]	No difference	12-month post-intervention	Group Exercise vs. Individual Physical Therapy
Limited	1 [29]	No difference	6-week post-randomization	Yoga vs. Conventional Therapeutic Exercise Classes vs. Self-care Book
Limited	1 [29]	No difference	3-month post-randomization	Yoga vs. Conventional Therapeutic Exercise Classes vs. Self-care Book
Limited	1 [29]	No difference	6-month post-randomization	Yoga vs. Conventional Therapeutic Exercise Classes vs. Self-care Book
**Functional Rating Index**				
Limited	1 [21]	No difference	Post-intervention	Exercise Group vs. Individual Treatment
Limited	1 [21]	No difference	6-month post-intervention	Exercise Group vs. Individual Treatment
**Participant Satisfaction Reporting Scale**				
Limited	1 [21]	No difference	Post-intervention	Exercise Group vs. Individual Treatment
Limited	1 [21]	No difference	6-month post-intervention	Exercise Group vs. Individual Treatment
**Pain Self-efficacy**				
Limited	1 [20]	No difference	3-month post-intervention	Group Exercise vs. Individual Physical Therapy
Limited	1 [20]	No difference	12-month post-intervention	Group Exercise vs. Individual Physical Therapy
Limited	1 [28]	No difference	6-month post-randomization	Group-based exercise + education vs. Cognitive functional therapy
Limited	1 [28]	A lower score for Cognitive functional therapy	12-month post-randomization	Group-based exercise + education vs. Cognitive functional therapy
**Risk of Chronicity**				
Limited	1 [28]	No difference	6-month post-randomization	Group-based exercise + education vs. Cognitive functional therapy
Limited	1 [28]	A lower score for Cognitive functional therapy	12-month post-randomization	Group-based exercise + education vs. Cognitive functional therapy
**Coping**				
Limited	1 [28]	No difference	6-month post-randomization	Group-based exercise + education vs. Cognitive functional therapy
Limited	1 [28]	A lower score for Cognitive functional therapy	12-month post-randomization	Group-based exercise + education vs. Cognitive functional therapy
**Number of Pain Sites**				
Limited	1 [28]	No difference	6-month post-randomization	Group-based exercise + education vs. Cognitive functional therapy
Limited	1 [28]	No difference	12-month post-randomization	Group-based exercise + education vs. Cognitive functional therapy
**Risk of Chronicity**				
Limited	1 [28]	No difference	6-month post-randomization	Group-based exercise + education vs. Cognitive functional therapy
Limited	1 [28]	No difference	12-month post-randomization	Group-based exercise + education vs. Cognitive functional therapy
**Sleep, Depression, and Anxiety**				
Limited	1 [28]	No difference	6-month post-randomization	Group-based exercise + education vs. Cognitive functional therapy
Limited	1 [28]	No difference	12-month post-randomization	Group-based exercise + education vs. Cognitive functional therapy
**Stress**				
Limited	1 [28]	No difference	6-month post-randomization	Group-based exercise + education vs. Cognitive functional therapy
Limited	1 [28]	No difference	12-month post-randomization	Group-based exercise + education vs. Cognitive functional therapy
**Satisfaction**				
Limited	1 [28]	No difference	6-month post-randomization	Group-based exercise + education vs. Cognitive functional therapy
Limited	1 [28]	No difference	12-month post-randomization	Group-based exercise + education vs. Cognitive functional therapy
**Short Form Health Survey–Physical Component**				
Limited	1 [20]	No difference	3-month post-intervention	Group Exercise vs. Individual Physical Therapy
Limited	1 [20]	No difference	12-month post-intervention	Group Exercise vs. Individual Physical Therapy
**Short Form Health Survey–Mental Component**				
Limited	1 [20]	No difference	3-month post-intervention	Group Exercise vs. Individual Physical Therapy
Limited	1 [20]	No difference	12-month post-intervention	Group Exercise vs. Individual Physical Therapy
**Increased work participation**				
Limited	1 [27]	No difference	12-month post-intervention	Brief Intervention vs. Brief Intervention + Cognitive Behavioral Therapy vs. Brief Intervention + Physical Group Exercise
**Hospitality Anxiety and Depression Scale**				
Limited	1 [27]	No difference	12-month post-intervention	Brief Intervention vs. Brief Intervention + Cognitive Behavioral Therapy vs. Brief Intervention + Physical Group Exercise
**Subjective Health Complaints Inventory**				
Limited	1 [27]	No difference	12-month post-intervention	Brief Intervention vs. Brief Intervention + Cognitive Behavioral Therapy vs. Brief Intervention + Physical Group Exercise
**Utrecht Coping List**				
Limited	1 [27]	No difference	12-month post-intervention	Brief Intervention vs. Brief Intervention + Cognitive Behavioral Therapy vs. Brief Intervention + Physical Group Exercise
**Instrumental Mastery-Orientated Coping**				
Limited	1 [27]	No difference	12-month post-intervention	Brief Intervention vs. Brief Intervention + Cognitive Behavioral Therapy vs. Brief Intervention + Physical Group Exercise
**Physical activity (International Physical Activity Questionnaire)**				
Limited	1 [24]	No difference	3-month post-randomization	Walking vs. Exercise Class vs. Usual Physiotherapy
Limited	1 [24]	No difference	6-month post-randomization	Walking vs. Exercise Class vs. Usual Physiotherapy
Limited	1 [24]	No difference	12-month post-randomization	Walking vs. Exercise Class vs. Usual Physiotherapy
**Exercise Self-efficacy Questionnaire**				
Limited	1 [24]	No difference	3-month post-randomization	Walking vs. Exercise Class vs. Usual Physiotherapy
Limited	1 [24]	No difference	6-month post-randomization	Walking vs. Exercise Class vs. Usual Physiotherapy
Limited	1 [24]	No difference	12-month post-randomization	Walking vs. Exercise Class vs. Usual Physiotherapy
**Readiness to Change Questionnaire**				
Limited	1 [24]	No difference	3-month post-randomization	Walking vs. Exercise Class vs. Usual Physiotherapy
Limited	1 [24]	No difference	6-month post-randomization	Walking vs. Exercise Class vs. Usual Physiotherapy
Limited	1 [24]	No difference	12-month post-randomization	Walking vs. Exercise Class vs. Usual Physiotherapy
**Patient Satisfaction Questionnaire**				
Limited	1 [24]	No difference	3-month post-randomization	Walking vs. Exercise Class vs. Usual Physiotherapy
**Left and Right Straight leg raising test**				
Limited	1 [23]	No difference	6-month post-randomization	Exercise Class vs. Individual Treatment
Limited	1 [23]	No difference	12-month post-randomization	Exercise Class vs. Individual Treatment
**Repeated sit-to-stand test/ Fifty-foot walk test/5-minute walk test/ Step-count for 1 Week**				
Limited	1 [26]	No difference	Post-intervention	Exercise &Education vs. Education
Limited	1 [26]	No difference	6-month post-intervention	Exercise &Education vs. Education
**Pain self-efficacy Questionnaire**				
Limited	1 [26]	More favourable results for the ED group	Post-intervention	Exercise &Education vs. Education
Limited	1 [26]	More favourable results for the ED group	6-month post-intervention	Exercise &Education vs. Education

## Results

### Studies included

The search identified 639 references after removing duplicates (Fig 1). Following title and abstract screening, 628 papers were excluded. One paper was identified by manual searching. This resulted in a total of 11 papers meeting the selection criteria. The most frequent reason for exclusion was inappropriate study design (e.g. did not carry out between-group comparisons).

### Pain information

Of the 11 studies meeting the inclusion criteria, all enrolled participants reported chronic LBP. All but one of the 11 studies reported on pain chronicity [20] (Table 2) Seven of the included studies reported pre-intervention and post-intervention pain intensity [20–26].

### Intervention used in the included studies

Table 3 summaries the intervention, duration, metric, and data collection time points used in the included studies. From the resulting 11 studies, 27 different outcome measurements were identified (Table 3).

### Methodological quality

Five studies met the methodological high-quality threshold of 70% (Table 4) [20, 22, 25, 28, 30]. Five studies scored between 60% and 69% [23, 24, 26, 27], and one scored 50% [21]. The major source of bias in the resulting 11 papers was the failure to formulate correlation and mean difference-testing hypotheses (i.e. a priori). These studies did not provide any information regarding the expected direction of correlations or if the mean differences met the original hypotheses. All studies clearly described 1) their sample size estimation for each experimental group and 2) their main findings.

### Measurement outcomes

From the resulting 11 studies, 47 different outcome measurements were identified with the resulting level of evidence and summary statements described in Table 5.

### Primary outcome measures

#### Self-administered disability measures

Low back pain associated disability was evaluated in 10 studies. Five studies used the Roland-Morris Disability Questionnaire [20, 25, 26, 29, 30]; four used the Oswestry Disability Index Questionnaire [22, 24, 27, 28] and one used Quebec back pain disability scale [23]. There was strong evidence of no difference between groups 3-month post-intervention from 3 high-quality studies and a study with moderate quality [20, 22, 26, 30]. Likewise, there was limited evidence of no difference between groups from one study for 9-month and 15-month post-intervention [20] and another study for 6-month post-randomization [24]. Two studies compared the post-intervention disability level with pre-intervention disability level [23, 26]. There was limited evidence of lower disability scores in people who received individual intervention compared to group exercise immediately and 6-month post-intervention. Results indicated limited evidence of no difference between exercise and education vs. education group only at 3-month and 6-month post-intervention compared to the base-line group [26]. The results were inconsistent from two studies 6-month post-intervention [23], from two studies 3-month post-randomization [24, 29], and three studies 6-month post-randomization [24, 28, 29]. There was limited evidence from one study for lower disability scores 4-week post-intervention (Table 5). People in the group exercise (intervention group) had a lower disability score than people in the waiting list (control) 4-week post-intervention [25]. Likewise, there was limited evidence from one study for lower disability scores 6-week post-randomization [29]. In this study, people in the yoga intervention group had a lower disability score than people in the booklet only group 6-week post-intervention [29]. In this study, the difference was not significant between yoga and conventional therapeutic exercise classes vs. self-care book, and between conventional therapeutic exercise classes vs. self-care book [29]. There was limited evidence from one study for lower disability scores 12-month post-randomisation (Table 5). Cognitive functional therapy led to greater reductions in disability compared with the group exercise intervention [28].

#### Pain

Pain level was measured in three studies using the Visual Analogue Scale [22, 23, 25] and using the Numeric Pain Rating Scale in four studies [21, 24, 26, 28] (Table 5). There was moderate evidence of no difference between groups for 6-month post-randomization and 12-month post-randomization [24, 28]. There was limited evidence of a lower pain score of people in the group exercise and education compared people of the education group 3-month and 6-month post-intervention compared to baseline [26]. There was limited evidence of non-difference between groups for immediately and 6-month post-intervention [21], 9-month and 15-month post-intervention [20], and 3-month post-randomization [24]. There was limited evidence of a lower pain score of people in the group exercise compared to people of the individual intervention group 4 week post-intervention [25].

### Secondary outcome measures

#### Quality of life

Quality of life was evaluated in four studies. Two studies used the EQ-5D quality of life scale [20, 30], one used the EQ-5D-5L, one used the EQ-VAS [30] and one study used the short form SF-36 Health Survey [29]. There was strong evidence of no difference between groups in health surveys scores from two high-quality studies [20, 30]. Likewise, there was limited evidence of no difference among groups for all measurement time points [20, 21, 29, 30].

#### Lumbar spine flexibility (flexion, extension, and lateral flexion)

There was limited evidence for no difference between groups post-intervention and 12-month post-intervention [23] with respect to group exercise vs. individual intervention on lumbar spine flexibility, however, there was limited evidence for more flexion, extension, and lateral bending range of motion in people of the group exercise group compared to the controls 4-week and 8-week post-intervention [25]. Likewise, there was limited evidence of a higher range of motion for lumbar extension and lateral bending 6-month post-intervention [23]. Differences in the flexion range of motion between these groups were not significant [23].

#### Fear beliefs

Low back pain associated fear beliefs were evaluated in three studies [24, 26, 27] with inconsistent results irrespective of the quality of the studies included. One study evaluated pain-related fear with the Tampa Scale of Kinesiophobia-13 (TSK-13, a modified version of the original Tampa scale of Kinesiophobia) [26], one used the Fear-avoidance Beliefs Questionnaire (FABQ) [27] and one used the Fear Avoidance Beliefs Questionnaire-PA subscale and Back Beliefs Questionnaire [24]. There was limited evidence of no difference among groups for fear beliefs 3-month post-intervention [24], 3-month and 6-mont post-randomization [26], either 6-month post-intervention [24] or post-randomisation [28], and either 12-month post-intervention [24, 27] or post-randomisation [28].

#### Other outcome comparisons

Most studies reported outcome measures in addition to those describing disability, quality of life and pain (Table 5). One study showed limited evidence that cognitive functional therapy was superior in pain self-efficacy, risk of chronicity, and coping compared to group-based exercise [28]. The remaining other outcome measures had limited evidence of no difference between the group and individual programs (Table 5).

## Discussion

### Main findings

The present systematic review identified strong evidence of no difference in disability level and pain scores 3-month post-intervention in people with chronic low back pain group-based exercise compared with controls that underwent other non-pharmacologic interventions. We also identified moderate evidence of no difference between group exercise and cognitive functional therapy for 6-month post-randomization and 12-month post-randomization. We could not find any strong or moderate evidence for or against the use of group-based exercise in the rehabilitation of people with chronic LBP for other time-points and health measurement outcomes.

These findings are consistent with findings of a recent systematic review conducted by O'Keeffe et al. [8] that compared individual exercise to group exercise for all musculoskeletal conditions including LBP. O’Keeffe et al. [8] found that for disability and pain, no clinically significant differences were found between the group and individual physiotherapy including exercise for all musculoskeletal conditions. They also found seven studies that specifically related to LBP that also noticed no clinically significant differences in disability and pain when comparing group and individual physiotherapy involving exercise [8].

While our results suggest there is no difference between group exercise and non-pharmacological interventions, there was one study that demonstrated limited evidence that cognitive functional therapy was superior in self-administered disability measures 6 and 12-month post-randomization compared to baseline. The same study indicated that cognitive functional therapy was superior in pain self-efficacy, risk of chronicity, and coping compared to group-based exercise 12-month post-randomization compared to 6-month post-randomization [28].

Some secondary outcomes demonstrated interesting findings but were not frequently used in the included studies. These included fear-avoidance, QoL and cost. Based on one study investigated here, group-based exercise reduced fear-avoidance scores [32], improved quality of life measures compared to usual general practitioner care [20] and lowered costs [23]. Based on these studies, further exploration of these outcomes in relation to group-based exercise performance is warranted.

### Study limitations

This review solely included studies published in English, and no search was conducted of the grey literature. These two factors may have caused a potential bias in selecting relevant studies. As discussed previously, the papers identified here were highly heterogeneous which prevented meta-analysis. Unfortunately, the literature was not sufficiently rich to focus our review on head-to-head comparisons of group-based exercise with individual-based exercise and other specific interventions.

Further, in terms of our specific summary statements, some of these studies conflicted with each other depending on the time-points compared (Table 5). The majority of conflicts were observed for timepoints with two or three studies (each study weighted 50% or 33.33% in the summary statement, respectively). This indicates that even a different observation from a low-quality study could drastically change the level of evidence for a specific summary statement. The limited evidence summary statements often showed no difference among interventions. The studies compared were heterogeneous in terms of the population studied (different ages, different time points, different pain and disability level among participants) or because of other methodological considerations, which may have contributed to the frequent conflicting evidence summary statements and limited our ability to observe consistent effects of group-based exercise.

## Conclusion

We identified strong evidence of no difference between group exercise and other non-pharmacological LBP interventions for disability level, quality of life, and pain. The remaining evidence was not of sufficiently high quality to permit further conclusions. With this equivocal finding, group-based exercise may be a preferred choice given potential advantages in other domains not reviewed here such as motivation and cost. Further research in this area is needed to evaluate this possibilty.

## Supporting information

S1 Checklist PRISMA 2009 checklist. (DOC)

S1 Appendix Library search keywords. (DOCX)

S2 Appendix Systematic literature review data extraction form. (DOCX)

S3 Appendix Appraisal form. (DOCX)

S4 Appendix (DOCX)
