# Supplementary material for: Comparing the effectiveness of group-based exercise to other non-pharmacological interventions for chronic low back pain: A systematic review
Source: PLoS One. 2020 Dec 30;15(12):e0244588. doi: 10.1371/journal.pone.0244588 (PMC7773269; doi:10.1371/journal.pone.0244588)
Supplement: S1 Appendix — (DOCX) [file pone.0244588.s002.docx]

**S1 Appendix:**

Library search keywords

***Ovid MEDLINE(R) and Epub Ahead of Print, In-Process & Other Non-Indexed Citations and Daily 1946 to July 23, 2019***

***Date searched: June 26, 2020***

***Results: 206***

1. (Good adj (life or living) adj2 osteoarthritis adj Denmark adj (back or lumbar)).mp.

2. (("GLA:D" or GLAD) adj8 (back-pain or lbp or lumbar-pain)).mp.

3. happy-back.mp.

4. ((Group* not (control-group* or intervention-group* or between-groups)) adj5 (exercise* or strengthening or physical-activity or strength-training)).mp.

5. ((chronic or persistent or long-standing or long-duration or ((duration or lasting) adj months)) and (back-pain or lbp or lumbar-pain)).mp.

6. 4 and 5

7. 1 or 2 or 3 or 6

***EMBASE 1974-July 23 2019***

***Date searched: June 26, 2020***

***Results: 280***

1. (Good adj (life or living) adj2 osteoarthritis adj Denmark adj (back or lumbar)).mp.

2. (("GLA:D" or GLAD) adj8 (back-pain or lbp or lumbar-pain)).mp.

3. happy-back.mp.

4. ((Group* not (control-group* or intervention-group* or between-groups)) adj5 (exercise* or strengthening or physical-activity or strength-training)).mp.

5. ((chronic or persistent or long-standing or long-duration or ((duration or lasting) adj months)) and (back-pain or lbp or lumbar-pain)).mp.

6. 4 and 5

7. 1 or 2 or 3 or 6

***CINAHL Plus with Full Text (EBSCOhost platform)***

***Date searched: June 26, 2020***

***Results: 262***

S1: (Good N1 (life or living) N2 osteoarthritis N1 Denmark N1 (back or lumbar)) OR (("GLA:D" or GLAD) N8 (back-pain or lbp or lumbar-pain)) OR happy-back

S2. ((Group* NOT (control-group* or intervention-group* or between-groups)) N5 (exercise* or strengthening or physical-activity or strength-training)) AND ((chronic or persistent or long-standing or long-duration or ((duration or lasting) N1 months)) AND (back-pain or lbp or lumbar-pain))

S3. S1 OR S2

***Scopus***

***Date searched: June 26, 2020***

***Results: 506***

TITLE-ABS-KEY ((Good W/1 (life or living) W/2 osteoarthritis W/1 Denmark W/1 (back or lumbar)) OR (("GLA:D" or GLAD) W/8 (back-pain or lbp or lumbar-pain)) OR happy-back) OR TITLE-ABS-KEY ((Group* W/5 (exercise* or strengthening or physical-activity or strength-training)) AND ((chronic or persistent or long-standing or long-duration or ((duration or lasting) W/1 months)) AND (back-pain or lbp or lumbar-pain)))

Key words

GLA:D

Back

Lumbar

Back pain, LBP, lumbar-pain

Happy-back

Group

Exercise, Strengthening, physical-activity, Strengthening-training

Chronic, persistent, long-standing, long-duration
