# Supplementary material for: Comparing the effectiveness of group-based exercise to other non-pharmacological interventions for chronic low back pain: A systematic review
Source: PLoS One. 2020 Dec 30;15(12):e0244588. doi: 10.1371/journal.pone.0244588 (PMC7773269; doi:10.1371/journal.pone.0244588)
Supplement: S2 Appendix — (DOCX) [file pone.0244588.s003.docx]

**S2 Appendix:** **SYSTEMATIC LITERATURE REVIEW DATA EXTRACTION FORM**

**Click here to choose a reviewer**

**Click here to enter a date.**

**Study description**

| ID No. |  |
| --- | --- |

**STUDY SELECTION CRITERIA**

| **Inclusion** | **Exclusion** |
| --- | --- |
| English | Other languages |
| Group Exercise VS Usual Care | Conference Proceedings |
| Original study | Specific LBP |
| Low Back Pain | Case Series < 10 Subjects |
| Full text available | Case Studies |
|  | Systematic Reviews |
|  | Narrative review |
|  | Protocols |

**DECISION:**  Included  Excluded  Unclear

**Study goals, type and timeline**

| Goal of the study |  |
| --- | --- |
| Type of the study |  |
| Timeline of study | Click to select the timeline of the study |

**Subject selection criteria.**

| Inclusion Criteria? | Not reported  Unclear  Yes, Specify: |
| --- | --- |
| Exclusion Criteria? | Not reported  Unclear  Yes, Specify: |
| Subject recruitment | Random  Consecutive  Volunteers  Purposeful. |

**Groups (definition) or define reported group and subgroups**

| Group name: |  |  |  |
| --- | --- | --- | --- |
| No of participants |  |  |  |
| Age |  |  |  |
| Diagnosis |  |  |  |
| Pain duration |  |  |  |
| Pain intensity |  |  |  |
| Pain location |  |  |  |
| Height |  |  |  |
| Weight |  |  |  |
| Gender | # of male:  # of female: | # of male:  # of female: | # of male:  # of female: |

**Exercise information (copy as needed if multiple methods were employed)**

| Type of exercise |  |
| --- | --- |
| Duration |  |

**List of measurements outcomes (for every subgroup/time-point/measure)**

Mean:

SD:

SE:

Range:

Min:

Max:

Other, specify:

**Results of statistical test of differences within and between groups:**

Name of the test used:

Variables compared:

Groups or times compared:

P-value (for each comparison of interest):

Report of the mean difference and variability for each pairwise comparison:

**Results of statistical test of correlations between the effects of group exercise and other variables of interest:**

Name of the association test used:

Variables tested:

Group tested:

Association estimate (correlation coefficient, regression equations)

P-values:

**Results of statistical test for diagnostic accuracy**

Name of the diagnostic statistical test used:

Name of the gold standard test:

Name of the variable of interest:

Diagnostic test estimate:

P-value or confidence interval:
