# Supplementary material for: Comparing the effectiveness of group-based exercise to other non-pharmacological interventions for chronic low back pain: A systematic review
Source: PLoS One. 2020 Dec 30;15(12):e0244588. doi: 10.1371/journal.pone.0244588 (PMC7773269; doi:10.1371/journal.pone.0244588)
Supplement: S3 Appendix — (DOCX) [file pone.0244588.s004.docx]

**S3 Appendix: Appraisal Form**

| Criterion | Yes | No | NA | Comments |
| --- | --- | --- | --- | --- |
| **Subjects recruitment** | | | | |
| Are the characteristics of the participants included in the study clearly described? |  |  |  |  |
| Were the demographic characteristics of the sample reported for each group analyzed? |  |  |  |  |
| Were the subjects asked to participate in the study representative of the entire population from which they were recruited? |  |  |  |  |
| Were those subjects who were prepared to participate representative of the entire population from which they were recruited? |  |  |  |  |
| Was an attempt made to blind study subjects to the intervention they received? |  |  |  |  |
| Were study subjects randomized to intervention groups? |  |  |  |  |
| Were participants’ characteristics stable during research? |  |  |  |  |
| **Examiners** | | | | |
| Were the training and qualifications of the examiner(s) reported? |  |  |  |  |
| Was/were the examiner(s) blinded to the results of the comparator test when comparing different test measurements? |  |  |  |  |
| Was/were the rater(s) blinded to the results of previous measurements performed by the same or different examiner(s) (e.g. blinded to pre-enrollment condition)? |  |  |  |  |
| Was the randomized intervention assignment concealed from health care staff until recruitment was complete and irrevocable? |  |  |  |  |
| **Methodology** | | | | |
| Are the exposures/interventions of interest clearly described? |  |  |  |  |
| Was the sample size included in the analysis adequate? |  |  |  |  |
| Is replication of the assessment procedure possible? (description sufficiently detailed) |  |  |  |  |
| Are the distributions of principal confounders in each group of subjects to be compared clearly described? |  |  |  |  |
| Was there an adequate adjustment for confounding in the analyses from which the main findings were drawn? |  |  |  |  |
| **Outcomes** | | | | |
| Is the hypothesis/aim/objective of the study clearly described? Must be explicit  (Only focus on objective related to the study of the effect of group exercise). |  |  |  |  |
| Validity reported for the main outcome measure |  |  |  |  |
| **Handling Missing Data (Concurrent and Criterion Validity)** | | | | |
| Compliance acceptable in all groups (80% acceptable) |  |  |  |  |
| Was the percentage of missing items given (Only for the analysis of the effect of group exercise)? |  |  |  |  |
| Withdrawal/dropouts rate described and acceptable |  |  |  |  |
| Have the characteristics of participants lost to follow-up been described? |  |  |  |  |
| Was compliance with the intervention/s reliable? |  |  |  |  |
| Was there a description of how missing items were handled? |  |  |  |  |
| Were hypotheses regarding correlations or mean differences formulated a priori (i.e. before data collection)? |  |  |  |  |
| Was the expected direction of correlations or mean differences included in the hypotheses? |  |  |  |  |
| **Statistical Analysis** | | | | |
| Have actual probability values been reported (e.g. 0.035 rather than <0.05) for the main outcomes except where the probability value is less than 0.001? |  |  |  |  |
| Sample size described for each group |  |  |  |  |
| Were design and statistical methods adequate for the hypotheses to be tested? |  |  |  |  |
| Has confidence interval for pre- and post-intervention or change in outcomes from before to after intervention been reported? |  |  |  |  |
| Have effect sizes for outcomes been reported or can be computed by the reviewer? |  |  |  |  |
| **Results** | | | | |
| Are the main findings of the study clearly described? |  |  |  |  |
| Have all important adverse events that may be a consequence of the intervention been reported? |  |  |  |  |

**Overall Score:**  out of 32 ( %)
