# Supplementary material for: Comparing the effectiveness of group-based exercise to other non-pharmacological interventions for chronic low back pain: A systematic review
Source: PLoS One. 2020 Dec 30;15(12):e0244588. doi: 10.1371/journal.pone.0244588 (PMC7773269; doi:10.1371/journal.pone.0244588)
Supplement: S4 Appendix — (DOCX) [file pone.0244588.s005.docx]

**Pain**

| **Author** | **Group** | **Baseline** | **Post-Intervention** | **4 weeks** | **3 Months** | **6 Months** | **9 Months** | **12 Months** | **12 Month** | **15 Months** |
| --- | --- | --- | --- | --- | --- | --- | --- | --- | --- | --- |
| **Hurely** | **Exercise** | 5.52 (5.52-5.52) |  |  | 5.05 (4.48-5.62) |  |  | 4.86 (4.23-5.49) | 5.12 (4.48-5.76) |  |
|  | **Walking** | 5.52 (5.52-5.52) |  |  | 4.46 (3.87-5.06) |  |  | 4.08 (3.42-4.74) | 4.16 (3.49-4.83) |  |
|  | **Usual physiotherapy** | 5.52 (5.52-5.52) |  |  | 4.31 (3.74-4.89) |  |  | 4.51 (3.87-5.15) | 4.13 (3.48-4.78) |  |
| **Johnson** | **Group Exercise** | 44.9 (18.2) |  |  | 29.1 (24.5) |  | 26.1 (23.5) |  |  | 27.9 (26.1) |
|  | **Control** | 51.6 (22.9) |  |  | 35.3 (26.7) |  | 35.0 (28.4) |  |  | 36.4 (27.3) |
| **Masharawi** | **Exercise** | 4.00 (1.43) | 1.86 (0.82) | 1.50 (0.84) |  |  |  |  |  |  |
|  | **Control** | 3.91 (1.64) | 3.88 (1.54) | NP |  |  |  |  |  |  |
| **O'Keefe** | **Cognitive Functional Therapy** | 6.17 (2.17) |  |  | 2.91 (2.47) | 3.77 (2.72) |  | 4.31 (2.50) |  |  |
|  | **Control** | 5.69 (2.23) |  |  | 4.60 (2.39) | 4.44 (2.36) |  | 4.88 (2.74) |  |  |
| **Ryan** | **Education + Exercise** | 28.1 (20.4) | 23.9 (23.3) |  | 19.1 (18.9) |  |  |  |  |  |
|  | **Education** | 39.3 (26.2) | 8.4 (7.5) |  | 22.6 (30.8) |  |  |  |  |  |
| **Sahin** | **Exercise** | 5.69 ± 2.14 | 4.91 ± 0.11 |  | 3.60 ± 0.15 |  |  |  |  |  |
|  | **Control** | 6.52 ± 1.12 | 5.35 ± 0.11 |  | 4.31 ± 0.15 |  |  |  |  |  |

**Oswestry Disability Questionnaire**

| **Author** | **Group** | **Baseline** | **Post-Intervention** | **3 Months** | **6 Months** | **12 Month** |
| --- | --- | --- | --- | --- | --- | --- |
| **Harris** | Brief Intervention | 28.07 (12.60) |  |  |  | 21.83 (13.80) |
|  | Brief Intervention + Cognitive Behavioral Therapy | 28.74 (12.70) |  |  |  | 22.83 (15.38) |
|  | Brief Intervention + Group Exercise | 29.58 (13.29) |  |  |  | 17.45 (13.60) |
| **O'Keefe** | Cognitive Functional Therapy | 32.05 (12.55) | 16.15 (9.74) |  | 20.19 (15.46) | 21.07 (13.62) |
|  | Control | 33.51 (12.61) | 26.11 (13.96) |  | 28.49 (16.96) | 28.43 (16.00) |
| **Sahin** | Exercise | 54.50 ± 14.13 | 41.01 ± 0.59 | 36.13 ± 0.69 |  |  |
|  | Control | 55.65 ± 11.80 | 44.76 ± 0.59 | 39.93 ± 0.69 |  |  |

**Roland Morris Disability Questionnaire**

| **Author** | **Group** | **Baseline** | **Post-Intervention** | **4 weeks** |
| --- | --- | --- | --- | --- |
| **Masharawi** | Exercise | 14.21 (5.22) | 9.31 (5.80) | 7.42 (5.42) |
|  | Control | 14.93 (5.96) | 14.37 (5.77) | NP |
